# Analysis of long non-coding RNAs in neonatal piglets at different stages of porcine deltacoronavirus infection

**DOI:** 10.1186/s12917-019-1862-4

**Published:** 2019-04-11

**Authors:** Xiaoyu Tang, Tian Lan, Ruiting Wu, Zhihai Zhou, Yuqi Chen, Yuan Sun, Yaoyao Zheng, Jingyun Ma

**Affiliations:** 1 College of Animal Science, South China Agricultural University, Tianhe District, Wushan Road 483, Guangzhou, 510642 China; 2 Key Laboratory of Animal Health Aquaculture and Environmental Control, Guangzhou, Guangdong China

**Keywords:** Long non-coding RNA (lncRNA), Porcine deltacoronavirus infection, Functional enrichment, High throughput sequencing, Interaction network

## Abstract

**Background:**

PDCoV (Porcine Deltacoronavirus) is a novel porcine coronavirus that causes intestinal necrosis of piglets, thinning of the intestinal wall and severe villus atrophy in the small intestine. PDCoV is a highly contagious infectious disease characterized by diarrhea, dehydration and vomiting. It has been reported that lncRNA has a significant effect on viral replication and increased or decreased virulence. At present, there is almost no research on lncRNA related to PDCoV infection. With the development of the research, a large number of lncRNAs related to PDCoV infection have been discovered. Identifying the role of these lncRNAs in the infection process facilitates the screening of diagnostically significant biomarkers.

**Results:**

Using high throughput sequencing to screen differentially expressed long non-coding RNA (lncRNA) during PDCoV infection, we identified 99, 41 and 33 differentially expressed lncRNAs in the early, middle and late stages of infection, respectively. These lncRNAs were involved in glycolysis / gluconeogenesis, histidine metabolism and pentose and Chloroalkane and chloroalkene degradation pathway. We obtained expression data of miRNAs, lncRNAs and mRNAs during PDCoV infection and constructed and investigated an interaction network. The qRT-PCR validation results of 6 differentially expressed lncRNAs were consistent with RNA-Seq results.

**Conclusions:**

This study is the first to examine differentially expressed lncRNAs after PDCoV infection of piglets. These results can provide new insights into PDCoV infection and antiviral strategies.

**Electronic supplementary material:**

The online version of this article (10.1186/s12917-019-1862-4) contains supplementary material, which is available to authorized users.

## Background

Porcine deltacoronavirus (PDCoV) belongs to the newly identified deltacoronavirus genus in the Coronaviridae family. This family possesses single-stranded positive-strand RNA genomes contained in an nucleocapside, so PDCoV is an enveloped virus. Intestinal infections in pigs result in diarrhea, vomiting and dehydration and are highly lethal. In 2009, 17 cases of swine diarrhea were positive for PDCoV and were linked to the deltacoronavirus [1]. The virus appeared again in 2014 causing large-scale outbreaks of pig diarrhea in the United States [2]. A retrospective study in 2015 identified PDCoV from samples collected from four Chinese provinces between 2004 and 2014 indicated an infection rate of 6.5% [3]. Subsequently, Thailand, Laos, Vietnam and the Philippines successively reported the existence of PDCoV [4, 5].

PDCoV has a broad host range and interacts with the catalytic domain of receptor aminopeptidase N (APN) in cat, chicken and human cells through the S1 domain B of the S protein, even though PDCoV infections in animals other than pigs have not been reported [6]. Ectopic expression of porcine aminopeptidase N (pAPN) in non-susceptible cells converted them to PDCoV-susceptible although pAPN is not a critical receptor but is an important accessory factor for infection [7]. PDCoV infection also affects the host’s innate immunity. PDCoV infection inhibited Interferon-beta (IFN-β) production by inhibiting nuclear factor-κB (NF-κB) and interferon regulation factor 3(IFN3) activation and inhibiting IFN-β promoter activation in the RIG-I pathway by suppressing IFN3 [8].

Long non-coding RNAs (lncRNA) are RNAs > 200 nucleotides in length with no protein encoding function. These RNAs play key roles in numerous biological functions as well as human disease. LncRNAs are involved as regulatory factors and are involved in epigenetic and transcriptional and post-transcriptional regulation. The functions of mammalian lncRNAs have been linked to metabolism, apoptosis and cell proliferation as well as immune responses including regulation of pattern recognition receptors and inflammation [9–12].

LncRNAs play crucial roles in host-virus interactions that include immune responsiveness and viral infections can induce their expression. For example, the differential expression of > 4800 lncRNAs were found in rhabdomyosarcoma cells after Enterovirus 71 infection [13]. In addition, > 3000 differentially expressed genes (DEG) were associated with lncRNAs during infectious salmon anaemia (ISA) virus infection and most were regulated in response to infection [14]. A total of 1236 lncRNA transcripts were differentially regulated at different stages of bovine viral diarrhea virus infected Madin Darby bovine kidney (MDBK) cells [15].

In the current study, we analyzed lncRNAs that were differentially expressed after PDCoV infection of piglets to examine whether lncRNAs are involved in PDCoV infection.

## Results

### Determination of tissue viral loads

**Fig. 1:**
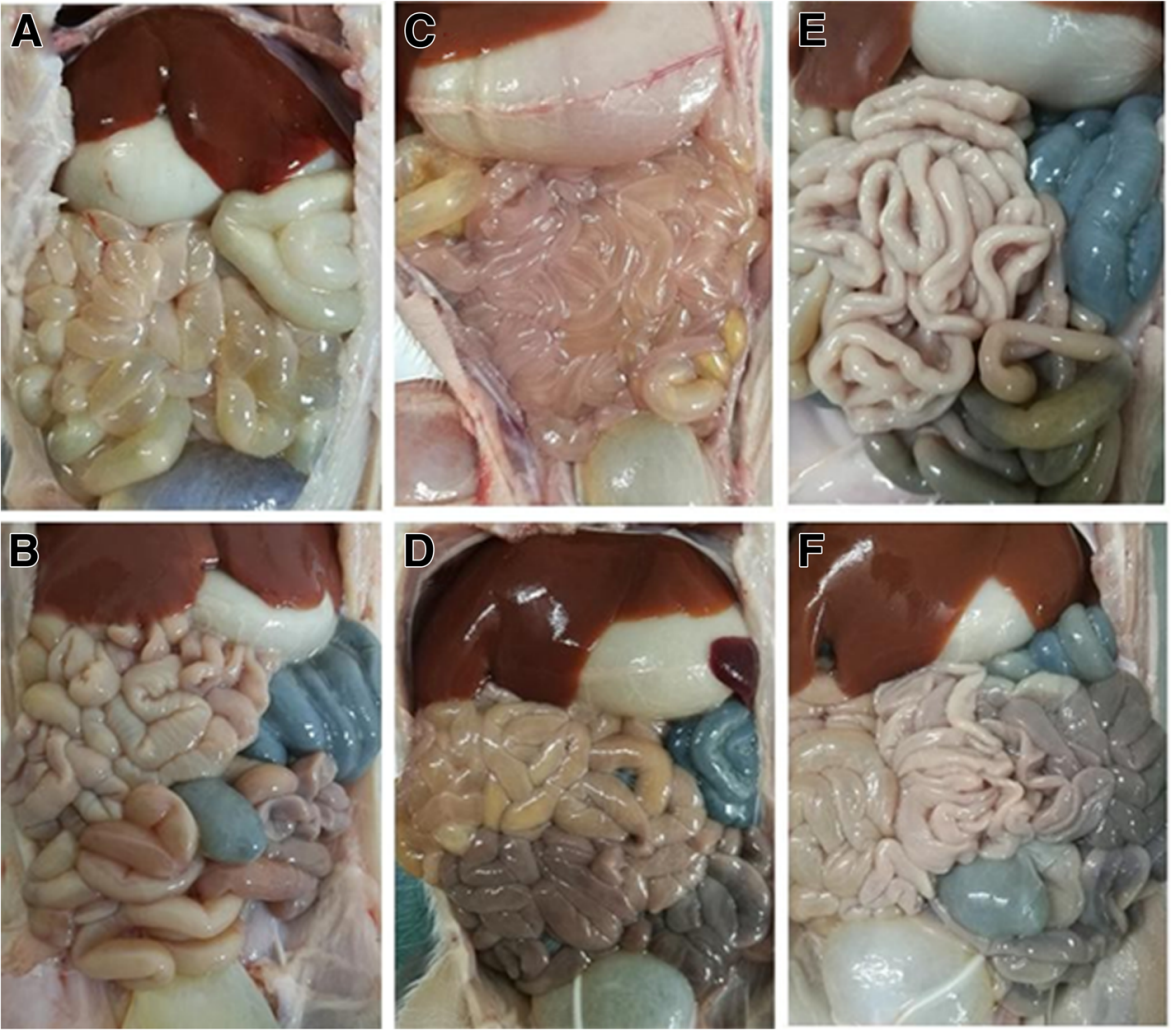
Abdominal anatomy of pigs at different infection stages of PDCoV infection. **a**, Experimental group at 2dpi; **b**, Control group at 2dpi; **c**, Experimental group at 4dpi; **d**, Control group at 4dpi; **e**, Experimental group at 11dpi; **f**, Control group at 11dpi

**Fig. 2:**
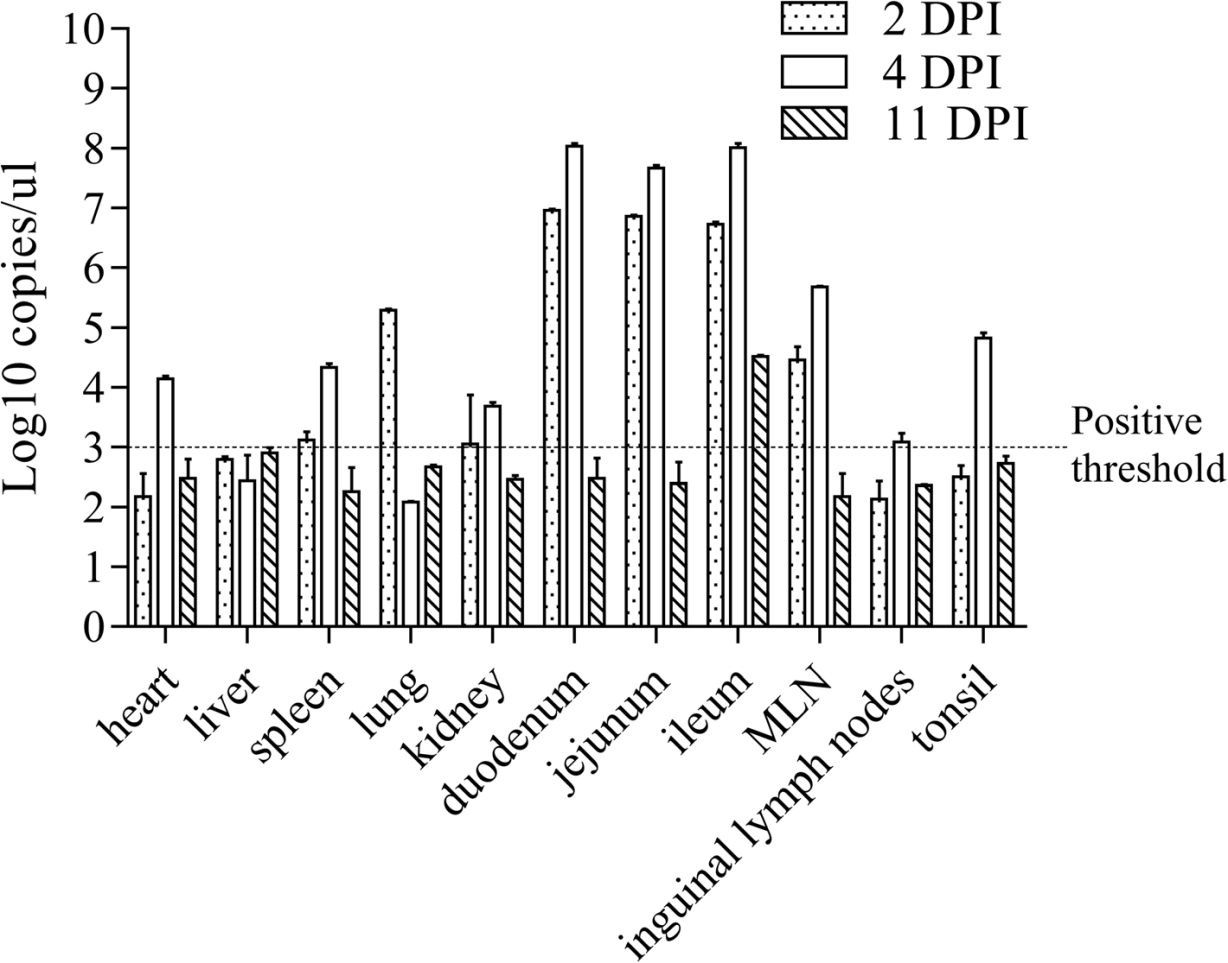
qRT-PCR results of tissue viral load after PDCoV infection in piglets

We plaque-purified the virus that we used for swine testicular cells (ST) cell infections and the virus with titer 1.0 × 10^8.6^ TCID50/ml was used for experimental infection of piglets. We found distinct differences in intestinal pathology between the experimental and the control groups at 2, 4 and 11 day post-infection (dpi) (Fig. 1). Piglet tissue samples taken at 2, 4 and 11 dpi were aseptically collected and the PDCoV RNA viral loads in each organ were determined by qPCR. The virus was found primarily in the small intestine. At mid-infection, viral loads in all organs except the kidney reached a maximum for all three periods. The heart, spleen, kidney, duodenum, jejunum, ileum, mesenteric lymph nodes and tonsils were all positive for PDCoV. At the late stage of infection, all organs except the ileum were PDCoV-negative (Fig. 2). The ileum was the most serious site of infection, so we used these samples for high throughput sequencing.

### Identification of lncRNAs in PDCoV -infected piglets by RNA-Seq

The samples we used for RNA-Seq were taken in the pre-onset (2 dpi), mid-onset (4 dpi) and late-onset (11 dpi). During the experiment, we chose three piglets from eighteen experimental piglets during the corresponding infection period for dissection, and the control group also dissected three piglets. The samples we used for RNA-seq were obtained from six piglets dissected at each stage. We analyzed the RNA-Seq data for 18 samples and each contained between 53,173,880 and 94,027,536 raw data points. After filtering, 52,830,072 to 93,138,526 clean data points were obtained for each sample and after assembly resulted in 2130 novel lncRNAs. In this group there were 173 differentially expressed lncRNAs identified in the three infection phases at a fold change > 2 and *P* < 0.05. The early, middle and late periods included 99 (21 up, 78 down), 41 (9 up, 32 down) and 33 (10 up, 23 down) lncRNAs, respectively (Table 1).

**Table 1:** Quantitative analysis of differential expression of lncRNA between experimental group and control group in different periods

Infection stage	Experimental group	Control group	Up-regulated lncRNAs	Down-regulated lncRNAs	Total differentially expressed lncRNA
Early	B1	A1	21	78	99
Middle	B2	A2	9	32	41
Late	B3	A3	10	23	33

### Target gene prediction and lncRNA analysis

LncRNAs are regulators of protein coding genes that lie near their genomic locations. We analyzed these RNAs for all protein coding genes within 100 kb of the lncRNA as potential cis-regulatory targets (Additional file 1).

Gene ontology (GO) analysis of DEGs indicated that 7, 7 and 9 terms were significantly enriched (*P* < 0.05) during early, middle and late infection periods, respectively. Molecular functions in the early period involved ferric-chelate reductase and oxidoreductase activity and metal ion oxidization. The middle period had terms related to oxidoreductase and prenyltransferase activity as well as positive regulation of activated T cell proliferation and catalytic activity. The late period terms were related to oxidoreductase activity, chromosome organization involved in the meiotic cell cycle and small molecule binding. Target genes related to oxidoreductase activity were found in all three periods (Additional file 2).

**Fig. 3:**
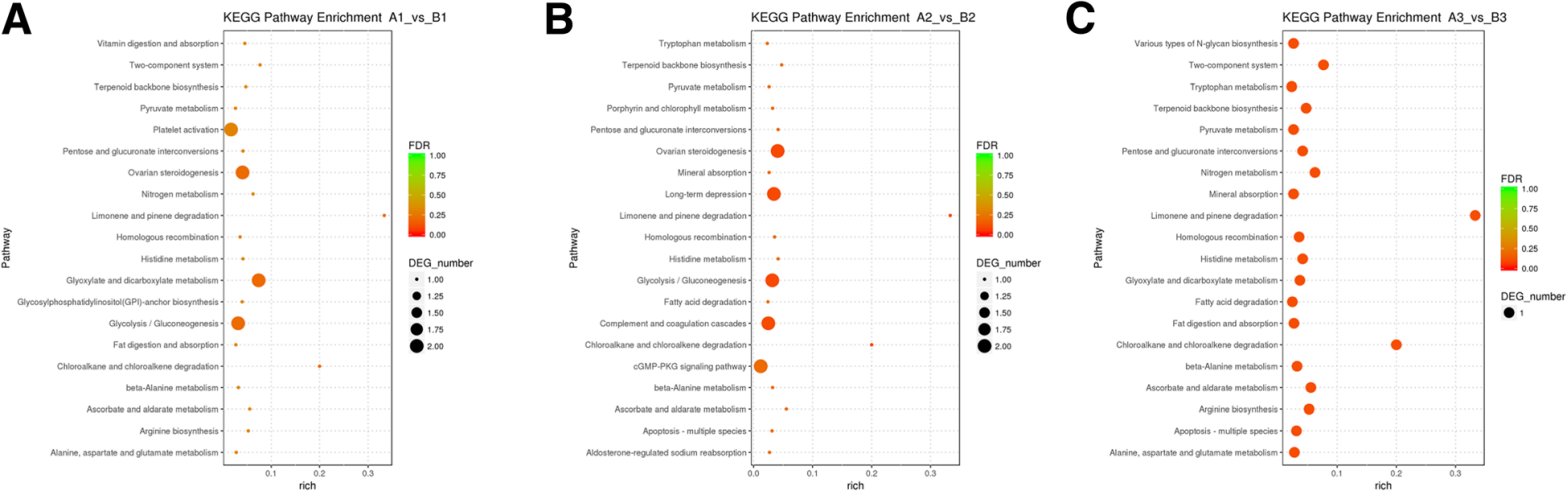
KEGG pathway enrichment analysis of target genes. **a**, The enrichment pathway of genes in the early infection stage; **b**, The enrichment pathway of genes in the middle infection stage; **c**, The enrichment pathway of genes in the late infection stage. Dot diameter is proportional to the number of differential genes and color depth is proportional to significance. Abscissa, enrichment ratio; ordinate, the different pathways. (The signal pathway shown in the figure that represents in the top 20 *p*-value ranking)

To elucidate the role of lncRNAs in the virus-host relationship, we performed Kyoto Encyclopedia of Genes and Genomes (KEGG) analysis of lncRNA target genes during each challenge period. We found that 6 pathways in the early period, 11 pathways in the middle and 14 pathways in the late period that were significantly enriched (*P* < 0.05). The temporal expression pattern of these pathways was related to both anabolic and catabolic functions. Anabolic functions included ascorbate and aldarate metabolism, histidine, β-alanine, glyoxylate and dicarboxylate metabolism. Catabolic pathways involved metabolism of limonene, pinene, chloroalkane and chloroalkene (Fig. 3a, b and c).

### lncRNA-miRNA-mRNA interaction network analysis

**Fig. 4:**
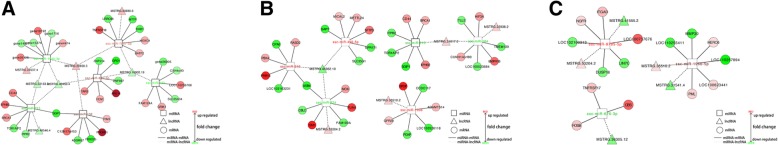
The lncRNA-miRNA-mRNA interaction network. **a**, pre-infection; **b**, middle stage and **c**, late stage of infection. Triangle, lncRNA; rectangle, miRNA; circle, mRNA. Red, up-regulation; green, down-regulation

CeRNAs (competitive endogenous RNA) are a class of functionally defined RNAs possessing miRNA binding sites that can compete with miRNA and inhibit their regulation of target genes [16]. Some lncRNAs in the network we constructed function as ceRNAs and we were able to construct a network based on these RNAs. The network was composed of 11 lncRNA nodes, 33 mRNA nodes and 10 miRNA nodes. The expression level of lncRNA is significantly up-regulated or down-regulated, and its target miRNA is down-regulated or up-regulated. The target gene expression level of miRNA is consistent with lncRNA. This suggests that there may be significant competitive RNA during infection. During the early infection stage, a total of 3 differentially expressed miRNAs were targeted by 4 lncRNAs and 9 mRNAs in the network. MSTRG.20658.3 and MSTRG.24537.4 can competitively bind to ssc-miR-194b-3p with mRNA. MSTRG.16902.19 and MSTRG.43450.4 can competitively bind to ssc-miR-885-5p and MSTRG.16902.19 can also competitively bind to ssc-miR-7857-3p (Fig. 4a). In the middle period, a total of 6 differentially expressed miRNAs were targeted by 5 lncRNAs and 18 mRNAs in the network. We found that MSTRG.45362.10 was capable of competitive binding to both ssc-miR-490-5p and ssc-miR-216, and MSTRG.31817.2 was capable of competitive binding to ssc-miR-215, ssc-miR-874 and ssc-miR-184. MSTRG.35510.2, MSTRG.22938.2 and MSTRG.53204.2 can competitively bind to ssc-miR-133b, ssc-miR-184, ssc-miR-874, respectively (Fig. 4b). In the late stage, a total of 2 differentially expressed miRNAs were targeted by 2 lncRNAs and 6 mRNAs in the network. MSTRG.31541.4 was capable of competitive binding to ssc-miR-196b-5p, and MSTRG.41555.2 can competitively bind to ssc-miR-9785-5p (Fig. 4c). The biological significance of ceRNA network in PDCoV infection was also reflected by topological structures including hubs and nodes as well as direct connections.

### Validation of differentially expressed lncRNAs

**Fig. 5:**
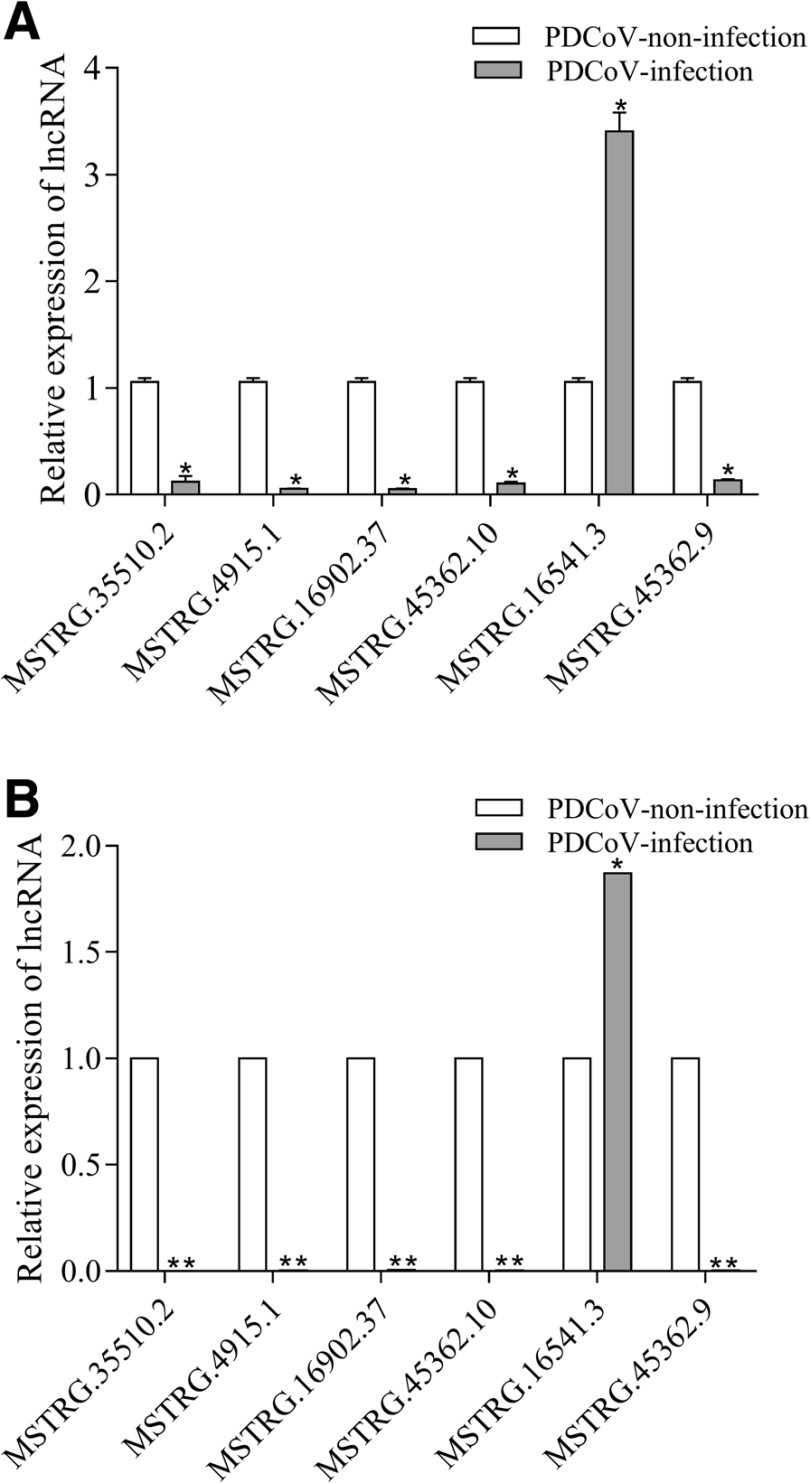
Verification of RNA-Seq results. Six differentially expressed lncRNAs in the middle stages of infection were randomly selected for real-time qRT-PCR experiments to verify whether their expression levels were consistent with the RNA-Seq results. **a**, the expression verification result of six lncRNAs (MSTRG.35510.2, MSTRG.4915.1, MSTRG.16902.37, MSTRG.45362.10, MSTRG.16541.3, MSTRG.45362.9); **b**, the RNA-Seq results of lncRNAs (MSTRG.35510.2, MSTRG.4915.1, MSTRG.16902.37, MSTRG.45362.10, MSTRG.16541.3, MSTRG.45362.9). (*n* = 3; ^*^p < 0.05, ^**^p < 0.01, ^***^p < 0.001)

The RNA-Seq analysis indicated that the expression level of many lncRNAs were significantly changed in the experimental group compared with the control group. In the mid-infection period, we randomly selected 6 lncRNAs (MSTRG.35510.2, MSTRG.4915.1, MSTRG.16902.37, MSTRG.45362.10, MSTRG.16541.3, MSTRG.45362.9) for expression level verification. Our validation results were consistent with the RNA-Seq results, so the results of RNA-Seq were credible. (Fig. 5).

## Discussion

PDCoV infects pigs of all ages, but primarily causes diarrhea in newborn pigs. Clinically, PDCoV infection is similar to porcine intestinal coronavirus, but PDCoV infection has a wider tissue tropism and can be detected in organs other than the digestive tract. This suggests a complex pathogenic mechanism for this virus and an in-depth understanding of its pathogenic and immune mechanisms is necessary for infection control.

Current lncRNA research has focused on human medicine including cardiovascular disease and cancer [17–19]. In livestock and poultry, examination of lncRNAs is in its infancy and existing research has focused on muscle, bone and embryonic development as well as fat metabolism [20–22]. Additionally, these studies have focused on lncRNA regulation of protein-coding genes.

Recent studies have shown that viral infections can induce lncRNAs to promote or inhibit viral responses. The lncRNA NEAT1 can up-regulate anti-HIV factors during infection and promote human immunodeficiency virus 1(HIV-1) replication [23]. The lncRNA ACOD1 enhances the replication of multiple viruses in both mouse and human cells [24]. However, an examination of lncRNA expression during PDCoV infections was lacking. The current study is the first to use comprehensive deep-sequencing technology that implicates lncRNAs in the response to PDCoV infection in pigs.

Our RNA-Seq data can assist in understanding the mechanism of action of differentially expressed lncRNAs at different stages of PDCoV infection. We established a lncRNA gene library that was generated during the early, middle and late stages of PDCoV infection. We identified 173 differentially expressed lncRNAs and 2130 novel lncRNAs. The greatest number of differentially expressed lncRNAs were found during the early stage of infection (2 dpi). The number of down regulated was >up-regulated lncRNAs. In addition, we found lncRNA MSTRG.18455 was significantly down regulated (− 7-fold) and its target gene IGF1 was significantly enriched. The insulin-like growth factor 1 (IGF1) is a member of the growth and development promoting signaling system and the main determinant of animal growth [25, 26].

Porcine enteroviruses can enter the digestive system through the mouth and subsequently attach to the intestinal villi. This causes villus atrophy resulting in diarrhea, dehydration, vomiting and weight loss. Our results from target mRNA pathway analysis revealed that pre-challenge target mRNAs of lncRNA were enriched for the signaling pathway of glyoxylate and dicarboxylate metabolism, limonene and pinene degradation, chloroalkane and chloroalkene degradation as well as glycolysis / gluconeogenesis. During the middle stages of infection, limonene and pinene degradation, glycolysis / gluconeogenesis, ascorbate and aldarate metabolism, histidine metabolism signaling pathway were prominent and late-stage target mRNAs were primarily concentrated on histidine metabolism, glyoxylate and dicarboxylate metabolism, beta-Alanine metabolism. It can be seen that some differentially expressed lncRNAs are related to the metabolism of organisms. The GO analysis indicated that at the middle stage of infection, these target genes were significantly enriched in the biological process category including regulation of activated T cell proliferation. Our future work will include determining lncRNA mechanisms of T cell activation and proliferation. This will assist in determining how the immune system is compromised by PDCoV infection in pigs.

In this study, we constructed a lncRNA-miRNA-mRNA interaction network containing a ceRNA network. Interestingly, both lncRNA and mRNA in the ceRNA network were negatively correlated with miRNA. Previous studies have confirmed that ceRNAs can act as miRNA ‘sponges’ and this is especially important for cancer and tumor diseases [27, 28]. By analyzing the miRNAs in the ceRNA network we found that the target miRNAs ssc-miR-885-5p, ssc-miR-490-5p, ssc-miR-196b-5p and ssc-miR-133b of lncRNAs were all up regulated. MiR-885-5p is a direct regulator of the IGF1 receptor that in combination with p73 regulate emergence of aggressive cancer stem-like features [29]. Decreased expression of miR-133b is associated with poor survival and increased metastasis in colorectal cancer [30]. miR-490-5p inhibits cell proliferation, migration and invasion but miR-490-5p can promote apoptosis of Human hepatoma (Hep3B) cells by inhibiting Roundabout Guidance Receptor 1 (ROBO1) [31]. MiR-196b-5p overexpression may also be associated with a risk of conversion of myelodysplastic syndrome (MDS) to acute myeloid leukemia (AML) [32]. The target miRNAs ssc-miR-194b-3p, ssc-miR-184, ssc-miR-215, ssc-miR-874 were all down regulated. miR-874 and miR-215 can act as a tumor suppressor [33, 34]. Therefore, this network plays a role in the expression of immune-related genes and the stimulation of host immune responses.

Our research provides a scientific reference for the lncRNAs that regulate PDCoV replication that can assist studies of ceRNA and PDCoV infection. This work can also aid the development of effective drugs and genetic engineering of pigs with PDCoV resistance.

## Conclusions

In this study, we provide the first analysis of differentially expressed lncRNAs after PDCoV infection in piglets. we constructed a lncRNA-miRNA-mRNA interaction network. This study provides insights into the relationships between lncRNAs and PDCoV immune modulation. Future studies will address lncRNA functions in immune escape used by PDCoV.

## Methods

### Cell culture and virus

ST cells used for experimental PDCoV infections in the study were kept in the Poultry Laboratory of the College of Animal Science, South China Agricultural University and cultured at 37 °C in a humidified 5% CO_2_ atmosphere in Dulbecco’s modified Eagle’s medium (DMEM, HyClone, Logan, UT, USA) supplemented with 10% fetal bovine serum (FBS, Hyclone) [35]. ST cells and culture media were checked to ensure the absence of PDCoV, porcine epidemic diarrhea virus (PEDV), transmissible gastroenteritis virus (TGEV) by gel electrophoresis of RT-PCR products. The virus strain PDCoV-CHN-GD16–05 (GenBank 74 accession no.KY363868.1) [36] was isolated and preserved by our laboratory from the watery diarrhea feces of nursing piglets in Guangdong Province, China. ST monolayers at 80% confluency were infected at a multiplicity of infection (MOI) of 1.5. The cells were cultured in serum-free medium at 37 °C for 1 h and the medium was replaced with fresh culture medium containing 2% FBS. Infected cells were collected 36 h post-infection (hpi).

### Determination of viral growth in tissues

The tissues we have taken include heart, liver, spleen, lung, kidney, duodenum, jejunum, ileum, mesenteric lymph nodes (MLN), inguinal lymph nodes and tonsils. Absolute quantification of viral RNA is done by real-time qRT-PCR. using the PCR primer pair 5′-TGGCTGATCCTCGCATCATGG-3’and 5′-GAGCGCATCCTTAAGTCT CTC-3′. One-step RT-PCR reactions were performed using an ABI PRISM 7500 (Applied Biosystems, Foster City, CA, USA) instrument according to the TAKARA company ‘PrimeScript™ RT reagent Kit with gDNA Eraser’ operating instructions. We used SYBR Green qPCR Super Mix (Invitrogen) for real-time qRT-PCR experiments based on amplification conditions. The following steps were used: denaturation at 95 °C for 10 mins and 40 cycles at 95 °C for 15 s, 60 °C for 30 s, 72 °C for 30 s and finally a melting curve.

### Piglet challenge experiments

The piglets were purchased from a farm in Huanong Wen’s Co., Ltd. Thirty 5-day-old piglets lacking any overt signs of infection were selected after observation for 24 h to ensure they were not exposed to stress before infection. The animals were infected with 5 mL of a virus solution containing 1.0 × 10^8.6^ TCID50/mL administered orally (18 piglets), and 12 control piglets were administered the same volume of DMEM. At the end of the experiment, the experimental animals showed weight loss, loss of appetite, and diarrhea, so they were euthanized. Experimental animal euthanasia method is based on the experimental animal management and practical technical manual. Intravenous injection of sodium pentobarbital at a dose of 90–100 mg/kg. Corpses were put into the septic tank, and they were fermented and used for fertilizer.

### RNA-Seq analysis

Three piglets were euthanized and dissected at the early (2 dpi), middle stage (4 dpi) and the late stages of infection period (11 dpi). Intestinal tissues with the most obvious lesions at each stage were collected in triplicate, frozen in liquid nitrogen and stored at − 80 °C. For controls, intestinal tissue was taken from control animals at similar time points.

Total RNA was extracted using Trizol reagent (Invitrogen) and purified using the Qiagen RNeasy Mini Kit (Qiagen, Valencia, CA, USA) according to the manufacturer’s instructions. RNA concentration and integrity were measured using the Agilent 2100 Bioanalyzer (Agilent Technologies, Palo Alto, CA, USA). We prepared RNA sequencing libraries from small intestine samples and performed 150-bp paired-end sequencing using the Illumina HiSeq platform. RNA sequencing libraries were prepared from 2 μg of total RNA using the TruSeq Kit (Illumina, San Diego, CA, USA) with the following modification. Instead of purifying poly-A RNA using poly-dT primer beads, we removed ribosomal RNA using the Ribo-Zero rRNA Removal Kit (Illumina). All other steps were performed according to the manufacturer’s protocol.

RNA-Seq libraries were quality control analyzed and the average insert size was 200 to 300 bp. The library was sequenced using a Hiseq platform (Illumina) at Shanghai Personal Biotechnology (Shanghai, China). Raw data was filtered and high-quality data was obtained. At the same time, the quality of the clean data was checked using the Q20, Q30 and GC content.

The Stringtie algorithm (https://ccb.jhu.edu/software/stringtie/) was used to analyze expression levels of lncRNA transcripts. The coding potential of candidate lncRNAs was analyzed to obtain high-confidence lncRNA using the Coding Potential Calculator (http://cpc.cbi.pku.edu.cn/), Coding-noncoding Index and Pfamscan (http://www.ebi.ac.uk/Tools/pfa/pfamscan).

LncRNAs are primarily located near genes with coding functions and the function of the associated lncRNA can be approximated by determining target gene function within 100 kb. GO (http://www.geneontology.org/) and KEGG (https://www.genome.jp/kegg) databases were used to determine functions and metabolic pathways of DEGs. GO terms and KEGG pathways with Q-values ≤0.05 were considered significantly enriched.

### Construction of lncRNA-miRNA-mRNA interaction network

LncRNAs, miRNAs and mRNAs that were differentially expressed between the experimental and control group tissues were chosen for analysis. The differential expression of miRNAs and lncRNAs was identified using standard selection criteria at *P* < 0.05 and fold change > 2. We used miRanda and psRobot software to predict target miRNA for lncRNA and target mRNA for miRNA (http://regrna2.mbc.nctu.edu.tw and http://miranda.org.uk) Target mRNAs were separately analyzed. The ceRNA network was generated from the interaction network and used Pearson correlation coefficient (PCC) to calculate the correlation between mRNA and lncRNA and chose pairs with positive correlations (PCC > 0.99 and P < 0.05). Visualization of the lncRNA-miRNA-mRNA interaction network was constructed using Cytoscape software (version 3.0; http://www.cytoscape.org/download.php).

### Screening and qRT-PCR validation of differentially expressed lncRNAs

Six lncRNAs (MSTRG.35510.2, MSTRG.4915.1, MSTRG.16902.37, MSTRG.45362.10, MSTRG.16541.3, MSTRG.45362.9) were randomly selected from the middle of the infection for real-time qRT-PCR validation, with glyceraldehyde-3-phosphate dehydrogenase (GAPDH) used as an endogenous control. Total RNA was extracted from uninfected and infected cells using a total RNA extraction kit (Tiangen Biotech, Beijing, China). cDNA was synthesized using a reverse transcription kit (Fisher Scientific, Pittsburg, PA, USA) with 2 μg of total RNA according to the manufacturer’s instructions. The primers were designed using Primer 5.0 software www.premierbiosoft.com (Table 2). One-step real-time RT-PCR reactions were performed using a One Step PrimeScript RT-PCR kit (Takara) on an ABI PRISM 7500 instrument. The following steps were used: reverse transcription at 42 °C for 10 min, denaturation at 95 °C for 10 s and 40 cycles at 95 °C for 5 s, 55 °C for 20 s, 72 °C for 10 s and finally a melting curve. Each reaction was performed in triplicate. LncRNA expression levels were calculated based on the 2 -ΔΔCT method [37]. Expression levels were normalized to those of GAPDH.

**Table 2:** DNA primers used for qRT-PCR

primers	Sequence (5′-3′)	amplicon
qPDCoV-F	TGGCTGATCCTCGCATCATGG	155 bp
qPDCoV-R	GAGCGCATCCTTAAGTCTCTC	
GADPH-F	ACATGGCCTCCAAGGAGTAAGA	150 bp
GADPH-R	GATCGAGTTGGGGCTGTGACT	
MSTRG.35510.2-F	CCACCAGCAACCAGGAACAGC	177 bp
MSTRG.35510.2-R	GCTCACAGCAACGCCAGATCC	
MSTRG.45362.9-F	TGCCGATTCCATTGTGCCATGAC	169 bp
MSTRG.45362.9-R	GTTGCTGTGGCTGTGGCTGTAG	
MSTRG.4915.1-F	ACTGTTGAAGCATGGCACAGA	90 bp
MSTRG.4915.1-R	TGTGGATGAAGGAACAGCAGG	
MSTRG.16902.37-F	AAGGAAGGTAACCGCAGGAGGAAG	107 bp
MSTRG.16902.37-R	GCTGCTGAGCTGAATTGCTAGGC	
MSTRG.45362.10-F	ATAGGAACCAGCAGGCGAGGAG	108 bp
MSTRG.45362.10-R	GGAGAGTGAGGAGGAAGGCAGTC	
MSTRG.16541.3-F	AGCAGTCAGAACCACCTGGAGAG	188 bp
MSTRG.16541.3-R	CACCACAGCTCACGGCAAGG	

### Statistical analysis

RNA-Seq data was analyzed using statistical R and expression levels of lncRNAs were compared using the paired sample t-test. Data were expressed as the mean ± standard deviation from at least three independent experiments. SPSS 17.0 software package (SPSS, Chicago, IL, USA) was used to analyze the qRT-PCR data. P < 0.05 was considered statistically significant. Statistical differences between the control and PDCoV infected cells were analyzed using the Student’s t-test.

## Additional files

Additional file 1: The target gene of lncRNAs. (XLSX 238 kb)

Additional file 2: Gene ontology (GO) analysis of target genes. (XLSX 10 kb)

## Abbreviations

AML: Acute myeloid leukemia
APN: Aminopeptidase N
CeRNA: Competitive endogenous RNA
DEG: Differentially expressed genes
DMEM: Dulbecco’s modified Eagle’s medium
dpi: days post-infection
FBS: Fetal bovine serum
GADPH: Glyceraldehyde-3-phosphate dehydrogenase
GO: Gene ontology
Hep3B: Human hepatoma
HIV: Human immunodeficiency virus
hpi: hours post-infection
IFN3: Interferon regulation factor 3
IFN-β: Interferon-beta
IGF1: Insulin-like growth factor 1
ISA: Infectious salmon anaemia
KEGG: Kyoto Encyclopedia of Genes and Genomes
lncRNA: Long non-coding RNA
MDBK: Madin Darby bovine kidney
MDS: Myelodysplastic syndrome
MOI: A multiplicity of infection
NF-κB: Nuclear factor-κB
pAPN: Porcine aminopeptidase N
PCC: Pearson correlation coefficient
PDCoV: Porcine deltacoronavirus
PEDV: Porcine epidemic diarrhea virus
ROBO1: Roundabout Guidance Receptor 1
ST: Swine testicular cells
TGEV: Transmissible gastroenteritis virus
